# Advances in CAR T cell immunotherapy for paediatric brain tumours

**DOI:** 10.3389/fonc.2022.873722

**Published:** 2022-11-23

**Authors:** Padmashree Rao, Liam Furst, Deborah Meyran, Chelsea Mayoh, Paul J. Neeson, Rachael Terry, Dong-Anh Khuong-Quang, Theo Mantamadiotis, Paul G. Ekert

**Affiliations:** ^1^ Translational Tumour Biology, Children’s Cancer Institute, Randwick, NSW, Australia; ^2^ Department of Microbiology & Immunology, The University of Melbourne, Victoria, VIC, Australia; ^3^ Murdoch Children’s Research Institute, Royal Children’s Hospital, Melbourne, VIC, Australia; ^4^ Cancer Immunology Program, Peter MacCallum Cancer Centre, Melbourne, VIC, Australia; ^5^ Sir Peter MacCallum Department of Oncology, University of Melbourne, Melbourne, VIC, Australia; ^6^ Université de Paris, Inserm, U976 Human Immunology Pathophysiology Immunotherapy (HIPI) Unit, Institut de Recherche Saint-Louis, Paris, France; ^7^ Children’s Cancer Centre, Royal Children’s Hospital, Parkville, VIC, Australia; ^8^ School of Women and Children’s Health, University of New South Wales, Randwick, NSW, Australia; ^9^ Department of Surgery Royal Melbourne Hospital (RMH), The University of Melbourne, Parkville, VIC, Australia; ^10^ Department of Paediatrics, University of Melbourne, Parkville, VIC, Australia

**Keywords:** CAR T cell, immunotherapy, paediatric brain tumour, tumour microenvironment, blood brain barrier

## Abstract

Brain tumours are the most common solid tumour in children and the leading cause of cancer related death in children. Current treatments include surgery, chemotherapy and radiotherapy. The need for aggressive treatment means many survivors are left with permanent severe disability, physical, intellectual and social. Recent progress in immunotherapy, including genetically engineered T cells with chimeric antigen receptors (CARs) for treating cancer, may provide new avenues to improved outcomes for patients with paediatric brain cancer. In this review we discuss advances in CAR T cell immunotherapy, the major CAR T cell targets that are in clinical and pre-clinical development with a focus on paediatric brain tumours, the paediatric brain tumour microenvironment and strategies used to improve CAR T cell therapy for paediatric tumours.

## Introduction

Tumours of the Central Nervous System (CNS) account for more than 25% of childhood cancers (1). The mainstays of paediatric brain cancer treatment are surgery, chemotherapy, and radiation therarpy. However, there is emerging interest in the potential for various immunotherapeutic modalities to improve the often dismal treatment outcomes for patients with CNS cancers, and to minimise the toxic and debilitating effects of standard treatments (2–4). The successes of adoptive cell therapy (ACT), particularly in B-cell malignancies, is driving the search for similar therapeutic approaches in other solid cancer types, including malignancies of the CNS (5). Autologous T cells, engineered to express a receptor recognising a tumour-specific or tumour-enriched antigen (Chimeric Antigen Receptor or CAR) are a promising type of ACT (6). The components that are assembled to make the CAR include a single-chain variable fragment (scFv) that binds the target antigen, linked *via* a transmembrane domain to one or more intracellular T cell signalling domains (6). The binding of the CAR to the target antigen is MHC-independent, which distinguishes CAR T activation from normal T-cell activation. CAR T cells are directly cytotoxic to their targets (7). The co-stimulatory domains are also critical to CAR T activity and persistance, and modifications of even a single amino acid residue in these domains can have profound effects on CAR T function (8).

The application of CAR T cell therapy to treat CNS tumours is an emerging field. The challenges are many, including identifying suitable antigens expressed at sufficient levels and which are tumour specific or enriched, generating effective CARs, and determining the most effective methods of delivery across the blood-brain barrier. However, early reports of favourable individual responses have forged the way for significant developments in CAR T technology and suggest that many, if not all, of these challenges can be met (6, 9, 10). The ultimate test of utility is, appropriately, the efficacy demonstrated in clinical trials. Several early phase trials are in progress and in this review, we explore the status of CAR T cell trials for paediatric brain tumours, the efforts to improve CAR T efficacy and focus on the ways in which the tumour microenvironment can influence CAR T activity.

## CAR T cells in clinical trials for paediatric brain tumours

The predominant clinical experience using CAR T cell therapy for central nervous system cancer, has focused on glioblastoma (GBM), which is rare in children but the commonest primary brain cancer in adults, and one that is invariably lethal (11). Therefore, the CAR T are directed against antigens enriched in this disease, including IL13Rα2, HER2 and EGFR variants. However, large scale genomic sequencing of paediatric cancers, including CNS cancers, have emphasised the distinct molecular nature of paediatric brain cancers (12–14). The clinical benefits that can flow from the appreciation of the specific driver events in individual tumours applies as much to immunotherapy as it does to targeted therapy using small molecule inhibitors (15). Thus, as one considers the important early clinical trials with CAR T directed against antigens such as IL13Rα2, HER2, and EGFR (**Table 1**), the lessons learned from these trials may be applied in the development of CAR-T specific, for the most challenging brain cancers which occur in children, including Histone 3.3 mutant paediatric glioma (16, 17).

**Table 1:** Clinical trials testing CAR T cell therapy in paediatric brain tumours.

Target	NCT Trial number	Trial Description	Tumour types	Age	Mode of delivery
**IL13Rα2**	04510051	Lymphodepletion followed by IL13Rα2 CAR T	Glioma, Ependymoma, Medulloblastoma, Germ Cell Tumour, ATRT, Primitive Neuroectodermal Tumour, Choroid Plexus Carcinoma, Pineoblastoma	1 to 26 years	Intraventricular
**IL13Rα2**	02208362	Genetically modified CAR T cells in recurrent or refractory malignant glioma.	High grade glioma	12 to 75 years	Tumour or tumour resection cavity
**GD2**	04099797	C7R-GD2 CAR T Cells GD2-expressing HGG,DIPG or MB (GAIL-B)	Embryonal tumour, HGG or ependymal tumour with confirmed GD2-expression (or H3K27M+ for HGG)	1 to 18 years	Tumour or tumour resection cavity
**GD2**	04196413	GD2 CAR T for H3.3 mutated DMG or DIPG	H3-K27M-mutated DIPG or DMG of the spinal cord	2 years to 30 years	Systemic
**HER2**	02442297	HER2-specific CAR for HER2-Positive CNS Tumours (iCAR)	Primary CNS tumour or HER2 positive tumour metastatic to the CNS (exclusion of DIPG)	3 years and older	Tumour, tumour resection cavity or ventricular system
**HER2**	03500991	HER2-specific CAR T Cell Locoregional Immunotherapy for HER2+ Recurrent/Refractory Paediatric CNS Tumours	Glioma, Ependymoma, Medulloblastoma, Germ Cell Tumour, ATRT, Primitive Neuroectodermal Tumour, Choroid Plexus Carcinoma, Pineoblastoma (exclusion of DIPG)	1 year to 26 years	tumour resection cavity or ventricular system
**HER2**	01109095	CMV-specific Cytotoxic T Lymphocytes Expressing CAR Targeting HER2 in Patients with GBM (HERT-GBM)	Glioblastoma	Child, adult,	systemic
**EGFR**	03638167	EGFR806-specific CAR T Cell Locoregional Immunotherapy for EGFR+ Recurrent or Refractory Paediatric CNS Tumours	Glioma, Ependymoma, Medulloblastoma, Germ Cell Tumour, Atypical Teratoid/Rhabdoid Tumour, Primitive Neuroectodermal Tumour, Choroid Plexus Carcinoma, Pineoblastoma (exclusion of DIPG)	1 year to 26 years	tumour resection cavity or ventricular system
**B7-H3**	04185038	Study of B7-H3-Specific CAR T Cell Locoregional Immunotherapy for Diffuse Intrinsic Pontine Glioma/Diffuse Midline Glioma and Recurrent or Refractory Paediatric Central Nervous System Tumours	Diffuse Intrinsic Pontine Glioma, Diffuse Midline Glioma, Ependymoma, Medulloblastoma, Germ Cell Tumour, Atypical Teratoid/Rhabdoid Tumour Primitive Neuroectodermal Tumour, Choroid Plexus Carcinoma, Pineoblastoma, Childhood Glioma	1 year to 26 years	tumour resection cavity or ventricular system

Table data were searched at clinicaltrials.gov (21^st^ June 2021).

### Interleukin-13 receptor alpha 2

The rationale for CAR T targeting of IL13Rα2 has been reviewed elsewhere (18, 19), but fundamentally stems from the specific over-expression of this antigen in GBM but not in healthy brain tissue. Preclinical mouse models showed that IL13Rα2 CAR T could cause regression of adult glioma patient-derived xenografts (PDX), including tumours derived from intracranial injection of a stem-cell enriched population (20). A current Phase I clinical trial (NCT04510051) is investigating the side effects of chemotherapy and cellular immunotherapy for the treatment of IL13R α2-positive recurrent or refractory brain cancer in children. An early study in adult patients with malignant glioma, using donor T-cells modified to express an IL-13 CAR–zetakine/HyTK CAR, administered together with interleukin-2, established the safety of the approach (NCT01082926). Further, encouraging results were published in an adult patient treated this time with autologous IL13Rα2 CAR T as part of a clinical trial investigating the efficacy of IL13Rα2 CAR T cells, open to patients over the age of 12 with recurrent or refractory glioma (NCT02208362) (6). These CAR are hinge-optimized, containing the 41BB-costimulatory domain as well as a truncated CD19 domain that permits tracking of the CAR T cells. Enrollment criteria include immunohistochemical evidence of IL13Rα2 protein expression in at least 20% of cells in the sample. In contrast to the systemic delivery of CAR T in B-cell malignancies, the CAR T are delivered directly into the tumour or into the ventricles of the brain. This highlights one of the distinct features of CAR T trials in CNS malignacies discussed later, relating to the potential for the blood brain barrier to limit CAR T access to tumours, and the potential efficacy of direct delivery of CAR T cells.

### Disialoganglioside

Gangliosides are glycolipids widely expressed, so are unlikley to be useful antigens for CAR T targeting. However, the disialoganglioside (GD2) has more restricted neuroectodermal expression, and is highly expressed in several cancers. From the paediatric cancer perspective, this was first reported in neuroblastoma, where a monclonal antibody generated against the LAN-1 neuroblastoma cell line was shown to recognise GD2 in neuroblastoma patient samples (21). Clinical trials using anti-GD2 monoclonal antibody therapy in neuroblastoma suggests this is more effective than standard treatments, at least in some clinical settings (22, 23). It is now appreciated that GD2 is also expressed in some paediatric brain tumours, notably diffuse midline gliomas (DMG) harbouring histone H3 K27M mutations, and in preclinical models a CAR T targeted against GD2 could effectively access the CNS and cause tumour regression (24). Currently, there at least two open trials of anti-GD2 CAR T cell therapy for paediatric patients with high grade glioma (HGG) and diffuse midline glioma (DMG), a subtype of DMG located in the brainstem (NCT 04099797 and NCT04196413). A recent breakthrough publication reports results from the first four patients with H3 K27M-DMG receiving a GD2 CAR T cell trial (25). The toxicity, which in some cases was significant, was mainly attributed to inflammatoiry effects and the tumour location. Three out of four patients derived radiographic and clinical benefit, but all patients ultimately succumed to the disease, despite persistance of CAR T cell activity. Interestingly, in preclinical models of anti-GD2 CAR T in neuroblastoma, persistence of neuroblastoma cells with low GD2 expression was observed (24), suggesting a possible escape mechanism.

### Human epidermal growth factor receptor

The Human epidermal growth factor receptor (HER) family is comprised of four transmembrane growth factor receptors; EGFR (HER1), ERBB2 (HER2), ERBB3 (HER3) and ERBB4 (HER4). HER2 is an established immunotherapeutic target in breast and ovarian cancer. In paediatric maligancies, HER2 alterations – principally copy number gains and gene over expression - was observed in 6% of all tumours in a paediatric pan-cancer cohort, predominatly in CNS tumours including Diffuse Hemishperic Gliomas (DHG), medulloblastoma, ependymoma, and a subset of neuroblastoma (14).

A phase I trial of HER2 specific CAR T cells in patients with HER2-positive GBM (NCT01109095) concluded that the therapy was safe, although the mean age of this cohort was 49 years, and only 6 paediatric patients were included. Of the 16 cases, eight showed an objective response, including one partial response (tumour regression) and seven patients with stable disease (26). Whilst the patient numbers were small, the results were encouraging, and the treatment was safe, paving the way for futher trials.

Currently, HER2 CAR T cells are being tested in a clinical trial for children with HER2^+^ brain tumours, over the age of 3 years (NCT02442297). The delivery method, as for the IL13Rα2 studies, was either by direct injection into the tumour resection cavity or by intraventricular delivery. An ongoing trial currently recruiting patients between the ages of 1 and 26 with HER2^+^ brain tumours, will test HER2 CAR T cell therapy, delivered *via* an indwelling catheter in the tumour resection cavity or the ventricles (NCT03500991). Interim analysis of the first three patients, one with localised anaplastic astrocytoma and two with metastatic ependymoma, confirmed that treatment was feasible without dose-limiting toxicity, and that a local immune response was activated by the CAR T cells. Two patients progressed shortly after initiation of treatment (27).

### EGFR

The epidermal growth factor receptor (EGFR or HER1) is another ErbB family member (28) being targeted by CAR T. EGFR is normally activated by ligand binding which triggers homo- or heterodimerization with other family members. Dimerization induces transphosphorylation of intracellular receptor domains and initiates down stream signaling (29). The oncogenic functions of EGFR are a result of constitutive EGFR activation by gene amplification, structural variants or point mutation. The spectrum of EGFR variants in childhood brain tumours is significantly different compared to those reported in adult glioma. In adult GBM, the EGFRvIII variant, where exons 2-7 are deleted, is the most common activating mutation. In childhood high-grade glioma (HGG), EGFRvIII mutations are less common (30), with gene amplification events and activating insertions in the tyrosine kinase domain being more frequently detected. In childhood cancers, co-occuring EGFR mutations and inactivating PTEN mutations are rare (14, 31). There are several potential therapeutic approaches to inhibit EGFR activation, including small molecule inhibitors, monoclonal antibodies and CAR T cells. Both small molecule inhibitors and monoclonal antibodies have not shown significant efficacy (32, 33). A completed clinical trial in adults (NCT01454596) indicated that in most patients, CAR T could be administered safely (with one treatment-related mortality), but there were no objective responses and no significant improvement in progression free survival. Another Phase I clinical trial (NCT 03638167) using EGFR806-specific CAR T cells in children and young adults with recurrent or refractory EGFR-positive CNS tumours is currently recruiting patients. In contrast to the previously mentioned study in adult patients, this study does not include concommitant adminstration of chemotherapy.

### B7-H3

Human B7-Homolog 3, B7-H3 (also known as CD276) is, in a cancer context, an immune checkpoint factor which may dampen the adaptive immune response to tumours. There is considerable interest in the possibility that blocking monoclonal antibodies directed agains B7-H3 could enhance CD8+ and NK cell infiltration into tumours (34). Interestingly, immunohistchemical staining of Diffuse Midline Glioma (DMG) patient samples showed that B7-H3 expression is elevated, with diffuse membrane staining of tumour cells in most tissues (35). This sugggests that B7-H3 could also be a tumour-specific antigen targetable by CAR T. Indeed, B7-H3 protein is expressed on a wide range of paediatric cancers, including high grade gliomas and medulloblastoma (36). Monoclonal antibodies with high-specificity for tumour-expressed B7-H3 were used as the basis for the development of a CAR T product. In preclinical studies of immunodeficient mice injected with a range of human cancer cell lines to generate orthotopic models, they showed promising activity, including in DMG and medulloblastoma (36). Further, atypical teratoid/rhabdoid tumours (AT/RTs), typically associated with germline mutations in the SWI/SNF complex gene *SMARCB1* (37, 38), also almost universally express elevated B7-H3 (39). B7-H3 CAR T cells, when administered by loco-regional routes, rapidly cleared tumour cells from the brain. Strikingly, the CAR T cells were able to traffic out of the CNS and prevent tumour redevelopemnt when treated mice were rechallenged with the parental cell lines (39). The current clinical study (NCT 04185038) builds from these observations of the safety and feasibility of B7-H3-specific CAR T cell infusions delivered into the tumour or ventricles. Since this CAR T demonstrates preclinical activity against some of the most difficult to treat paediatric brain cancers with particularly dismal prognosis in the case of AT/RT, there is potential for significant clinical impact.

## Major challenges in CAR T cell therapy for CNS tumours

Factors common to all CAR T cell therapies may determine their efficacy, such as the antigen load and specificity in the target cells, the proliferative capacity of the CAR T cells, their persistence post delivery, the biological features of the transduced T cells and the nature of co-stimulatory signals included with the CAR (19, 40–43). More specific to cancers of the CNS are the challenges presented by delivery of CAR T cells across the blood brain barrier and the unique immunosuppressive nature of the brain tumour microenvironment (TME).

### The paediatric brain tumour microenvironment

**Figure 1:**
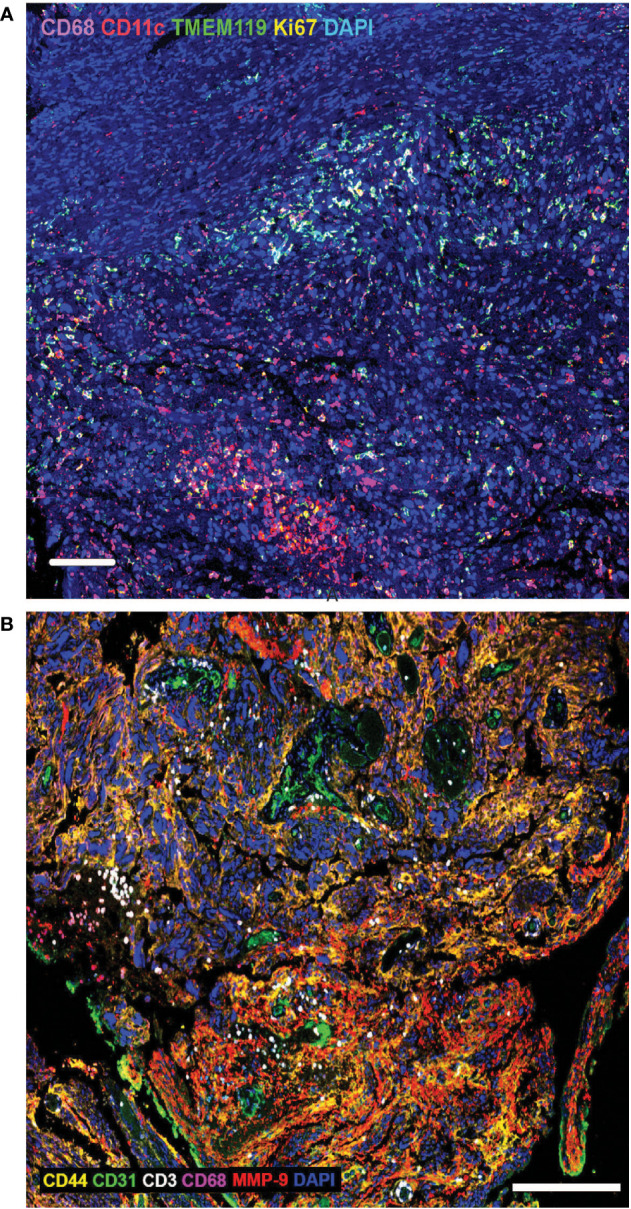
**(A)** Extensive heterogeneity and distinct spatial distribution of immune cells in paediatric brain tumour microenvironment. Multiplex immunohistochemistry analysis of paediatric high-grade glioma tissue using biomarkers to identify macrophages (CD68), dendritic cells (CD11c), microglia (TMEM119) and proliferating cells (Ki67). DAPI labels all cell nuclei. Tumour infiltrating immune cells are often present in clusters, with macrophages penetrating deeper into tumour cell-rich regions, compared with dendritic cells and microglia. Scale bar is 100*µ*m. **(B)** Extensive heterogeneity and distinct spatial distribution of T-cells and macrophages in adult brain tumour microenvironment. Multiplex immunohistochemistry analysis of adult glioblastoma tissue using biomarkers to identify tumour cells (CD44), endothelial cells/blood vessels (CD31), T-cells (CD3), macrophages (CD68), matrix metalloproteinase-9 (MMP9); DAPI labels all cell nuclei. Tumour infiltrating T-cells are often present in clusters, in close proximity to blood vessels, while macrophages penetrate deeper into tumour cell-rich regions. Scale bar is 200*µ*m.

Brain tumour cells thrive by adapting to their microenvironment. Infiltrating immune cells are among the major non-cancer cell types in tumours and the key factors influencing tumour biology and immunotherapeutic efficacy (44). The establishment of an immunosuppressive TME is a major contributing factor to failure of adoptive immunotherapies in solid tumours, preventing immune cell penetration of the tumours and quenching their activity. The brain is also a relatively immune privileged organ (45). Much of the current knowledge of the brain TME is based on adult brain tumours, and the paediatric brain TME biology has, until recently, been inferred from such studies even though it is well understood that many significant age-specific changes occur during pre- and postnatal development, and the immune system continues to develop until adulthood (46). There are also differences in blood brain barrier structure and function, as well as differences in remodelling of the brain TME in response to therapy. A common feature of both the adult and paediatric brain TME is the cellular and molecular heterogeneity (47). Using techniques such as multiplex immunohistochemistry, the ability to not only measure the cellular and molecular composition of the TME, but the ability to also interrogate the spatial relationship between cells and immunomodulatory factors is uncovering the functional biology of the TME (48). Investigation of paediatric high-grade glioma tissue using multiplex immunohistochemical highlights the TME cellular heterogeneity and shows the extent of immune cell infiltration and spatial distribution of the tumour infiltrating cells (TILs) in relation to histopathological hallmarks (**Figure 1A**) and in adult glioma tissue using multiplex immunohistochemistry (**Figure 1B**). A recent study suggests that the composition and extent of immune cell infiltration is a reliable prognostic indicator of adult GBM patient survival and predictive of patient response to chemotherapy (49). Paediatric gliomas also exhibit distinct spatial localisation of specific T-cell subtypes. TCF1+ T-cells, which are immature T-cells, were largely localised within perivascular niches, while CD103+ tissue-resident memory T-cells (T_RM_) were observed deep within tumour cell-rich regions (50). Whether the distribution of these T-cell subtypes will be important instructive in treatment of paediatric glioma remains to be seen, but studies in other cancers in adults, suggest that the presence of high tumour-specific T_RM_ cell number in breast cancer patients correlates with improved prognosis and longer overall survival and better response to anti-PD-1 immunotherapy in advanced-stage breast cancer patients (51). Thus, spatial analysis of TILs and the TME, with respect to the immune cell type, and the extent of infiltration into tumour cell-rich regions, will likely be an important characteristic of paediatric brain tumour biology and may correlate with response to immunotherapy, including CAR T therapy.

### Blood brain barrier

The blood-brain barrier is a key determinant of CAR-T access to CNS tumours. The blood brain barrier is formed by tight junctions between endothelial cells and is the first-line physical barrier that contributes to the immune privileged status of the CNS (52). While recent studies have shown that activated immune cells are able to traverse the blood brain barrier, their passage is significantly restricted (53). Disruptions to the blood brain barrier is a well characterized feature of CNS diseases, including multiple sclerosis (54) and ischemic stroke (55). Tumour growth is accompanied by progressive necrosis in the tumour core, triggering vascular endothelial growth factor (VEGF) secretion in the surrounding cells, and the generation of immature, leaky blood vessels (56). Vascular “leakiness” might be expected to facilitate efficient trafficking of CAR T cells into the tumour. Indeed, a clinical trial in adult GBM patients using EGFRvIII CAR T cells showed evidence of post-therapy tumour T-cell infiltration (57).

### Immunosuppression in the paediatric brain TME

In adult HGG, including GBM, immune cells can comprise up to 40% of the TME, including macrophages, microglia, and regulatory T-cells (58). Moreover, tumour cells and glioma cancer stem cells can recruit immunosuppressive macrophages *via* secretion of various immunosuppressive cytokines and growth factors (59). Broadly, the paediatric brain cancer TME is different with respect to the immune cell populations and the cytokine milieu. Immunoprofiling of paediatric low-grade glioma (LGG), DHG and DMG identified elevated numbers of immunosuppressive macrophages (CD163+) in LGG and DHG compared to non-tumour tissue, with a proportion of samples also having CD8+ T-cell infiltrates. In contrast, the DMG TME was largely devoid of CD163+ immunosuppressive macrophages and T-cells. These differences suggest that factors influencing the TME in glioma may include the distinct molecular features of these tumours and perhaps even the anatomical location (58, 60).

The complexity of the interactions between CNS tumour cells and surrounding and infiltrating cells are increasingly recognised as determinants of the effectiveness of immunotherapies, including CAR T cell therapies, and may be influenced by a myriad of factors (61). Moreover, the TME is a dynamic environment and may change over the course of treatment. Intriguing but preliminary observations of CSF myeloid cells in patients treated with anti-GD2 CAR T for DMG, suggests that the inflammatory nature of myeloid cells varies depending on the route of CAR T administration (intravenous compared to intraventricular) and the time of sampling, after treatment (25). T-cell infiltration in paediatric glioma is, to some degree, dependent on malignancy, with lower grade gliomas tending to show increased CD3+ T cell number compared to higher grade gliomas, although there is considerable heterogeneity (50). Immunosuppressive T-cell phenotype infiltration also tends to increase with disease progression (62). It is not known whether T-cell infiltration in some tumours is a response to specific tumour antigens, or what immunosuppressive mechanisms and factors are important in primary childhood CNS cancers. It may be that identification of immunogenic T-cell peptide antigens may be useful targets for future adoptive cellular therapies (63).

### CAR T cell toxicity

CAR T cell therapy has significant toxicities, the most common of which might be considered an “on-target” effect. The therapeutic goal of exciting a cellular immune response in the brain is a partiuclar concern. Cytokine release sydrome (CRS) is an inflammatory condition arising as a consequence of the activation of the infused CAR T by its target antigen. The inflammatory consequences include systemic symptoms such as fever, myalgia and rigors but may also prgress to more serious consequences include capilliary leak syndrome and consequent hypotension, circulatory collapse, and end-organ failure including neurotoxicity (64). Of the many cytokines detectable in the peripheral blood during CRS, Interleukin-6 levels correlate most closely with disease severity (65), and IL-6 inhibition is effective therapy for severe CRS (64). Interestingly, host antigen presenting cells and not the infused CAR T are the source of IL-6 in CRS (66). Neurological toxicity is a specific concern, at least in theory, for CAR T therapy for CNS cancers. The early clinical experinces from the phase I trials indicate that dose limiting toxicities have not yet been a major issue. Anecdotal experience from these studies clearly describes local inflammation after CAR T, which when it is in critical anatomical sites such as the brainstem, may have life-threatening consequences (25). Since the toxicity of CAR-T therapy derives from the on target activation of the CAR T, another way to control this is to limit the life span of the CAR T cells. So-called “suicide switches” are engineered into the CAR T. Most commonly, these involve a constuct encoding chimeric proetin with a dimerization domain and a functional domain which can induce CAR T cell death (such as a caspase-9). Whilst not yet features of current approved CAR T therapies, are an effective mechansim to clear the CAR T cells (67, 68). The trade-off is that killing the CAR T precludes any further therapeutic beneift. Finally, clinical observations in 9 patients with B-cell maliganacies treated with anti-CD19 CAR T suggests that there may be reduced toxicity associated infusion following low dose chemotherapy (69). It is unknown how prior therapy might influence the response to CAR T in CNS patients, except to not that all the phase I clinical trials currently active recruit heavily pretreated patients.

## Meeting the challenges for CAR T cell therapy for paediatric brain tumours

Beyond the identification of target antigens in paediatric brain tumours and the development of effective CARs, there are important therapeutic considerations to ensure effective trafficking of CAR T cells to the brain, either by direct inoculation to the tumour site or with the use of targeted combination therapy to overcome the immunosuppressive brain tumour microenvironment. Some of these therapeutic strategies are discussed below.

### The blood brain barrier

Several strategies have been used to deliver CAR T cells across the BBB, most prominently by direct introduction into the tumour resection cavity or into the ventricular system of the brain.

The direct innoculation of CAR T to the tumour site is an obvious strategy to bypass tumour-intrinsic or anatomical barriers, including the BBB, and immunosppressive niches which restrict traffiking of CAR T within tumours following systemic adminstration (70). Moreover, direct tumour innoculation may diminish on-target side effects of CAR T therapy by limiting the exposure of normal tissue expressing the target antigen to the CAR T cells (71). Both efficacy and safety considerations, together with clear evidence from preclinical studies (9), underpin the use of direct deleivery of CAR T cells to into the tumour resection cavity or ventricular system (NCT 03500991, NCT 03638167, NCT 04185038). Another potential approach is to modify the BBB so that it presents a less challenging barrier for CAR T passage into the brain. For example, Sabbagh et al. showed low intensity pulsed ultrasound could enhance tumour infiltration of systemically delivered CAR T, in a preclinical xenograft model of glioma (72). Radiation therapy prior to CAR T adminstration also appears to an effective mechanism to provide CAR T cells with access beyond the BBB (73). Although it is beyond the scope of this review, there are many molecular and physical differences between the BBB in the tumour microenvironment compared to normal tissue (reviewed in (74)). Some of these may resent opportunities for intervention to give systemically depvered CAR T access to their intracranial targets. However at this stage, more direct delivery options seem to be the best option.

### Combination therapy

Vascular endothelial growth factor A (VEGFA) is a proangiogenic growth factor that functions as a ligand for VEGF Receptor 2 (VEGFR2). Blocking VEGFA antibodies also have an immunomodulatory effect. Stickingly, VEGFA-blocking antibodies significantly delayed tumour progression in a melanoma model (B16 cells) when combined with a CAR T engineered to recognize a tumour specific antigen. Notably, without the CAR T, the antibody had no effect on tumour progression, suggesting the effect was not solely the result of an antiangiogenic effect (75). Moreover, simultaneous targeting of the tumour specific antigen and VEGFR2 by CAR T cells caused tumour regression in the same model (76). It seems likely that the immunomodulatory effects of disrupting the VEGF-VEGFR axis is a result of both direct effects on immune cells and effects on tumour vaculature (77). Since the anti-VEGFA antibody Bevacizumab is approved for use in GBM, the potential of combining this agent with a CAR T in CNS tumour therapy is an intriguing possibility (78), but as yet, there are no published data in humans. Emerging clinical evidence in adult mesothelioma supports the principle of combining VEGFA inhibitors with checkpoint inhibitor drugs to activate TILs (79). Whether such combinations can extend to CAR T in CNS cancers in children is not known, but an intriguing and promising line of investigation. There is also likely to be further complexity in the brain since the VEGF signalling also regulate the BBB (74) and it is plausible that VEGF inhibition might reduce BBB permeability.

### Overcoming the immunosuppressive brain tumour microenvironment

There is interest in understanding whether existing drugs which inhibit immuno-suppresive checkpoints can facilitate the entry of CAR T into CNS tumours. Checkpoint inhibitors have had virtually no clinical impact in paediatric maligancies with the sole, but important, exception of hypermutated cancers arising in the context of germline mutations of mismatch repair genes (Mismatch Repair Deficiency – MMRD), with or without Polymerase Proofreading deficiency (PPD). In chiildhood, a significant proportion of such patients present with gliomas (80). These tumours have orders of magnitude more mutations than non-MMRD tumours, and as a consequence, a much larger number of potential neoantigens. From this perspective, these missmatch repair mutated tumours resemble cancers with high tumor mutation burden, such as melanoma, which is one of the cancers most responsive to checkpoint inhibition. However, there is evidence to support the view that combining checkpoint inhibitor drugs with exogenously delivered CAR T may be an effective strategy, although the evidence is primarily derived from adult phase I trials in patients with malignant pleural disease, and not from patients with CNS cancer (81). A high proportion of T-cells in paediatric CNS tumours exhibit an exhausted phenotype and expression of PD-L1 (60). Observations that concomitant CAR T and anti-PD-1 therapy results in CAR T cell expansion, downregulated CAR T PD-1 expression and tumour regression, particularly in diffuse large B-cell lymphoma (DLBCL) (82), further supports the promise of this approach. However, the phase 1b PORTIA study testing tisagenlecleucel, a CD19 directed CAR T cell in combination with pembrolizumab, in patients with refractory or relapsed DLBCL did not report improved efficacy compared to tisagenlecleucel alone, in a small cohort of patients, leading to an early termination of the study (NCT03630159) (83). Alternative mechanisms to disprupt checkpoint signaling in CAR T prior to administration, including targeted gene deletion, have been reviewed elsewhere (84). For patients with GBM, a clinical study (NCT04003649) is evaluating the safety and feasability of a combination therapy of IL13Rα2-specific CAR T cells alone, or in combination with the immune check point inhibitors, nivolumab or ipilimumab.

## Conclusion

Immunotherapy in cancer treatment is having a renaissance, with checkpoint inhibitor therapy for melanoma and lung cancer, and CAR T cell therapy for B-cell malignancies showing promising results (5, 85, 86). The successes for these diseases raises the question on how immunotherapy can be improved to provide longterm anti-tumour responses? The challenges presented by maligancies of the CNS in children and in adults, are considerable, and as yet, are largely unrealised. However this should not be taken to mean that these challenges are insurmountable. From the CAR T perspective, the search for new CNS-tumour specific antigens against which CARs can be generated is supported by the remarkable increase in the genomic and transcriptomic profiling of paediatric cancers [reviewed in Jones et al. (87)]. Moreover, there are many potential innovative strategies to alter the CNS tumour microenvironment with existing drugs including checkpoint inhibitors and kinase inhibitors, and the ability to modify CAR T cells directly to enhance traffiking to and activity within the CNS. Perhaps the most important endevours are those seeking to understand the fundamental basis of the immune environment of the CNS, and how the cancer cells interact with the non-cancer cells, including tumour infiltrating immune cells. A more fundamental appreciation of the immune mechanisms that permit CNS tumours to escape immune survellence will provide the knowledge base for the development novel and successful immune-based therapies.

## Author contributions

All authors have contributed to the conception and writing of this review. All authors have read and approved the final version of the manuscript. All authors contributed to the article and approved the submitted version.

## Funding

PE acknowledges the support of Perpetual Trustees and the Samuel Nissen Foundation, as well as the Steven Walter Children’s Cancer Foundation. This research was supported by an Australian Government Research Training Program (RTP) Scholarship (CM). The Medical Research Future Fund through the Emerging Priorities and Consumer Driven Research scheme and the Australian Brain Cancer Mission/National Health & Medical Research Council/Lifting Clinical Trials and Registry Capacity (NHMRC MRF9500002) for salary support (CM).

## Conflict of interest

The authors declare that the research was conducted in the absence of any commercial or financial relationships that could be construed as a potential conflict of interest.

## Publisher’s note

All claims expressed in this article are solely those of the authors and do not necessarily represent those of their affiliated organizations, or those of the publisher, the editors and the reviewers. Any product that may be evaluated in this article, or claim that may be made by its manufacturer, is not guaranteed or endorsed by the publisher.
